# Predicting seasonal influenza transmission using functional regression models with temporal dependence

**DOI:** 10.1371/journal.pone.0194250

**Published:** 2018-04-25

**Authors:** Manuel Oviedo de la Fuente, Manuel Febrero-Bande, María Pilar Muñoz, Àngela Domínguez

**Affiliations:** 1 Technological Institute for Industrial Mathematics (ITMATI), Campus Vida, Santiago de Compostela, Spain; 2 MODESTYA Group, Department of Statistics, Mathematical Analysis and Optimization, Universidade de Santiago de Compostela, Campus Vida, Santiago de Compostela, Spain; 3 Department of Statistics and Operation Research, Universitat Politècnica de Catalunya, Barcelona, Spain; 4 CIBER en Epidemiología y Salud Pública (CIBERESP), Madrid, Spain; 5 Department of Medicine, Universitat de Barcelona, Barcelona, Spain; Universidad Miguel Hernandez de Elche, SPAIN

## Abstract

This paper proposes a novel approach that uses meteorological information to predict the incidence of influenza in Galicia (Spain). It extends the Generalized Least Squares (GLS) methods in the multivariate framework to functional regression models with dependent errors. These kinds of models are useful when the recent history of the incidence of influenza are readily unavailable (for instance, by delays on the communication with health informants) and the prediction must be constructed by correcting the temporal dependence of the residuals and using more accessible variables. A simulation study shows that the GLS estimators render better estimations of the parameters associated with the regression model than they do with the classical models. They obtain extremely good results from the predictive point of view and are competitive with the classical time series approach for the incidence of influenza. An iterative version of the GLS estimator (called iGLS) was also proposed that can help to model complicated dependence structures. For constructing the model, the distance correlation measure R was employed to select relevant information to predict influenza rate mixing multivariate and functional variables. These kinds of models are extremely useful to health managers in allocating resources in advance to manage influenza epidemics.

## Introduction

Influenza is an infectious disease with person-to-person transmission that characteristically occurs as an epidemic affecting the whole population [1]. The influenza virus has been categorized into types A, B and C. However influenza C is a mild disease without seasonality and is therefore not considered in influenza epidemics. One remarkable feature of the influenza A and B viruses is the frequency of changes in antigenicity. Alterations in the antigenic structure of the virus leads to infection by variants to which the population has little or no immunity.

The epidemiology of inter-pandemic influenza (also named seasonal influenza) is characterized in temperate zones by epidemics of variable size that occur during the colder winter months (November to April in the Northern Hemisphere and May to September in the Southern Hemisphere), each of which typically lasts 8–10 weeks [2]. In a study on influenza activity throughout eight seasons (1999–2007), the average length of epidemics in 23 European countries was 15.6 weeks (median 15 weeks; range 12–19 weeks) [3].

The reasons for the seasonal presentation of influenza epidemics are not entirely clear but they might result from more favourable environmental conditions for virus survival [4]. Various theories including improved virus survival in low temperatures, low humidity and low levels of ultraviolet radiation [2] have been advanced to explain this pattern in temperate zones. The typical incubation period for influenza is 1–4 days (average: 2 days).

Surveillance systems require accurate indicators that detect possible epidemics in advance. The epidemic of influenza is one of the problems of most concern to public health professionals across the world, due to its high levels of mortality and morbidity. Influenza is highly contagious and causes more morbidity than any other vaccine-preventable illness [5]. So, accurate estimates of the incidence of influenza are essential, for both public health services and citizens, to provide advance warning of epidemics and allow preventive measures to reduce contagion.

Statistical methods to forecast the incidence of influenza in particular, and contagious diseases in general, have changed over time. In one of the first studies on time series, Choi and Thacker [6] employed an ARIMA model to estimate pneumonia and influenza mortality. Dushoff et al. [7] used a regression model to investigate how cold temperatures contribute to excess seasonal mortality. Hohle and Paul [8] proposed an alternative model to monitor infectious diseases that consisted in applying count data charts to monitor time series. From a Bayesian framework, Conesa et al. [9] an automated monitoring of influenza surveillance data that made it possible to take the geographical component into account in statistical models in addition to temporal evolution was proposed. Contributions to this methodology are growing steadily through disease mapping. The studies by Ugarte et al. [10] and Paul and Held [11] are recent examples of this. Their common denominator is that they apply different statistical methodologies to multivariate time series (hierarchical Bayesian space–time, mixed models, P–splines and conditional autoregressive models -CAR-, among others) of infectious disease counts, collected in different geographic areas, using multivariate or longitudinal data.

Functional data analysis (FDA) has grown in popularity over recent years alongside the increasing availability of continuous measurements in different contexts like Biomedicine [12], Spectrometry [13], Biology [14] and Medicine [15], to mention only a few. This study extends the regression models for independent functional data to the case where the curves presents either spatial or temporal dependencies.

Our goal is to estimate the rate of influenza epidemics, using the information readily available from public sources possibly that include functional variables, by adapting or extending the GLS techniques from a multivariate framework to this new framework. So, our particular aim is to estimate dependence components of influenza, using regression models, and predict the rate of incidence of influenza for a horizon of two weeks. We initially model influenza using a traditional linear approach (with independent errors) and later extend these ideas to the functional case (with dependent errors).

The article is structured as follows. Methodology section presents the Generalized Least Squares (GLS) approach for functional regression models. The estimation of the different parameters (for the regression function or the dependence) is usually done using maximum likelihood although, as an alternative, we introduce an iterative GLS (iGLS) procedure that provides similar results. The latter could be interesting when the structure of the dependence is complicated. The practical performances of the GLS and iGLS procedures are compared, by means of a simulation study. Real example section applies these models to the prediction of the influenza rate in a region of Spain.

## Methodology

The functional regression model (FRM) is one of the most studied topics in FDA over the last few years. A regression model is said to be “functional” if any of the variates involved (the predictors or the response) has a functional nature, i.e. it is a measure observed along a continuous interval. Cases with a scalar response and functional predictors have particularly attracted a lot of attention. For example, Sørensen et al. [15] gives a basic introduction for the analysis of functional data applied in datasets from medical science.

The functional regression model with scalar response (FRM) is stated as follows: Let X,y two random variates taking values in E×R where E is a functional space (semi-metric, normed or Hilbert). The relationship between the two variates can be expressed as follows:

$$\begin{array}{c} y=m(X)+\epsilon =E(y|X)+\epsilon \end{array}$$ (1)

where *ϵ* is a real random variable verifying E(ϵ|X)=0. Depending on the nature of the functional space E and on the regression operator *m*, we can classify the different types of FRM:

- **Multivariate Linear Model**: $E=R^{p}$ and *m* is the linear operator in the space, i.e. E(y|X)=Xβ with $\beta \in R^{p}$.
- **Functional Linear Model**: $E=L^{2}\left(T\right)$ is the Hilbert space of square integrable functions over *T* = [*a*, *b*] and *m* is a linear operator in the space, i.e. m(X)=〈X,β〉 with $\beta \in L^{2}\left(T\right)$. This model has been treated extensively in the literature mainly devoted to the optimal way of representing the linear operator through the representation of X and *β* on a basis of $L^{2}\left(T\right)$.
  Depending on the latter, the references can be classified into two main categories:
  - Fixed basis. The most commonly used basis in this context are the Fourier [16], the B-spline [17] and the Wavelet [18].
  - Data-driven basis. Two main basis computed from the data are used in the literature: the most parsimonious one is given by the functional principal components [19, 20] and the one that maximizes the covariance among the response and the functional predictor uses the functional partial least square components (PLS) [21, 22].
  Note that, due to the representation employed, the FRM is always an approximated model and its goodness typically relies on the properties of the chosen basis and its suitability to the data at hand.
- **Functional Non Linear Model**: E is (at least) a semi-metric space and *m* is a continuous operator i.e. ${lim}_{X^{\prime }\to X}m\left(X^{\prime }\right)=m\left(X\right)$. For a complete review of this model see Ferraty and Vieu [13] and the references therein.
- **Extensions of the above models**: The above models could be extended in several ways, usually considering more than one predictive variate. This could lead to semi-linear models [23, 24], additive models [25, 26], [27], single index models [28, 29] or projection pursuit models [30].

Many of the above-mentioned authors consider that *ϵ* = (*ϵ*_1_, …, *ϵ*_*n*_)′ is an homoskedastic independent error vector, i.e. E(ϵ)=0, $V\text{ar}(\epsilon )=\sigma^{2}$ and Cov(*ϵ*_*i*_, *ϵ*_*j*_) = 0, *i* ≠ *j*. This assumption is made to obtain simple diagnostics or confidence intervals for the response but it could be too restrictive in functional regression models and difficult to check or fulfill in practice. Some papers consider dependence in the functional variate. See, for example, [31, 32] and [33] for contributions devoted to spatial dependence with functional data or [34–36] and [37] for time dependence. In both cases, the functional nature of the variate complicates the predictive ability of the model. The aim of this paper is to extend the GLS approach [38] to the functional context as the simplest way of incorporating temporal or spatial dependence in the regression models. In fact, the GLS approach can handle a wide range of regression models with dependence in a simple way: equi-correlation models, random effects, time and spatial dependence, and so on. This idea was first introduced in the context of FDA in [39].

### Functional generalized least squares regression

The functional generalized least squares regression (FGLS) model between two centered variables (E(y)=0, E(X)=0) states that

$$\begin{array}{c} y=\left\langle X,\beta \right\rangle +\epsilon =\int_{T}X\left(t\right)\beta \left(t\right)dt+\epsilon \end{array}$$ (2)

where $\beta \epsilon L_{2}\left(T\right)$ and *ϵ* is now a random vector with mean 0 and covariance matrix $\Omega =E(\epsilon \epsilon^{\prime })$. This model includes, as its special cases, many others models, all of them based on Ω = Ω(*ϕ*) = *σ*^2^ Σ(*ϕ*), where *ϕ* is the parameter associated with the dependence structure of Ω. Some classical examples are presented in the following models:

1. Equi-correlated model: $V\text{ar}(\epsilon_{i})=\sigma^{2}$ and Cov(*ϵ*_*i*_, *ϵ*_*j*_) = *σ*^2^ *ϕ*, *i* ≠ *j*, *ϕ* ∈ (−1, 1)
2. Heteroskedastic block model: $\Omega =diag\left(\sigma_{1}^{2}{\text{I}}_{n_{1}}|\sigma_{2}^{2}{\text{I}}_{n_{2}}|\cdots |\sigma_{p}^{2}{\text{I}}_{n_{p}}\right)$ with *n*_1_ + *n*_2_ + ⋯ + *n*_*p*_ = *n*
3. AR(1) model: *ϵ*_*i*_ = *ϕϵ*_*i*−1_ + *ε*_*i*_ with |*ϕ*| < 1, $E(\varepsilon_{i})=0$, $V\text{ar}(\varepsilon_{i})=\tau^{2}$ and Cov(*ε*_*i*_, *ε*_*j*_) = 0, *i* ≠ *j*
  $$\begin{array}{c} \begin{array}{c} \Omega =\frac{\tau^{2}}{1-\phi^{2}}{\left(\phi^{\left|i-j\right|}\right)}_{i,j=1}^{n} \end{array} \end{array}$$
  The variance structure is also known for every ARMA(*p*,*q*) model.
4. Spatial correlation model:
  $$\begin{array}{c} \Omega =\sigma^{2}(\rho (d(s_{i},s_{j}))) \end{array}$$
  where *s*_*i*_,*s*_*j*_ are, respectively, the locations for *i*, *j*; and *ρ* is the spatial correlation function.

#### Estimation of functional GLS

The classical theory of Kariya and Kurata [38] can be extended to the functional case by adapting the GLS criterion accordingly, i.e.

$$\begin{array}{c} GLS\left(\beta ,\phi \right)={\left(y-\langle X,\beta \rangle \right)}^{\prime }\Sigma {\left(\phi \right)}^{-1}\left(y-\langle X,\beta \rangle \right) \end{array}$$

Given the sample $\left\{\left(X_{1},y_{1}\right),\ldots ,\left(X_{n},y_{n}\right)\right\}$, we can approximate $X_{i}$ and *β* using a finite sum of the basis elements:

$$\begin{array}{c} X_{i}\left(t\right)\approx \sum_{k}^{K_{x}}c_{ik}\psi_{k}\left(t\right),\;\beta \left(t\right)\approx \sum_{k}^{K_{\beta }}b_{k}\varphi_{k}\left(t\right) \end{array}$$

The preceding equations can be expressed as matrix notation using the evaluation in a grid of the length *M* {*a* = *t*_1_ < ⋯ < *t*_*M*_ = *b*} as

$$\begin{array}{c} X=C\Psi ,\;\;B=b^{\prime }\varphi , \end{array}$$

where **X** is the matrix *n* × *M* with the evaluations of the curves in the grid, **C** is the matrix *n* × *K*_*x*_ with the coefficients of the representation in the basis and Ψ is the matrix *K*_*x*_ × *M* with the evaluations of the basis elements on the grid. Similarly, **B** is the matrix (1 × *M*) with the evaluation of the *β* parameter on the grid, *φ* is the matrix (*K*_*β*_ × *M*) with the evaluations of the basis {*φ*_*j*_} and **b** on the grid, is the vector of the coefficients of *β* in the basis.

With this notation, the terms ${\left\{\langle X_{i},\beta \rangle \right\}}_{i=1}^{n}$ can be approximated by **C** Ψ*φ*′ **b** = **Z** **b** which, in essence, is a reformulation of a classical multivariate linear model that approximates the functional model. Here, the matrix **Z** takes into account all the approximation steps done with the available information: the chosen basis for X and *β* with the selected components: *K*_*x*_ and *K*_*β*_.

Once a certain approximation is selected, supposing that *ϕ* is known, we can define **W** = Σ(*ϕ*)^−1^, and use the classical theory for multivariate GLS to obtain the BLUE of **b** through:

$$\begin{array}{c} b_{\Sigma }={\left(Z^{\prime }\text{WZ}\right)}^{-1}Z^{\prime }Wy, \end{array}$$

where **b**_Σ_ has covariance

$$\begin{array}{c} \text{Cov}(b_{\Sigma })=\sigma^{2}{\left(Z^{\prime }\text{WZ}\right)}^{-1} \end{array}$$

Finally, the fitted values are obtained by:

$$\begin{array}{c} \hat{y}=Z{\left(Z^{\prime }\text{WZ}\right)}^{-1}Z^{\prime }Wy=Hy \end{array}$$

where **H** is the hat matrix.

Once the model is estimated, we can compute the prediction for a collection of *m* new data $\left\{X_{0}^{j}\right\}$ using the model chosen for Σ. Being *ϵ*_0_ the vector of errors for the new points, Δ′ = Cov(*ϵ*, *ϵ*_0_) and $\Sigma_{0}=V\text{ar}(\epsilon_{\text{0}})$, we can obtain the equations for prediction:

$$\begin{array}{c} \hat{y_{0}}=\left\langle X_{0},\hat{\beta }\right\rangle +\Delta \Sigma^{-1}(y-\langle X,\hat{\beta }\rangle ) \end{array}$$

$$\begin{array}{c} V\text{ar}({\hat{y}}_{0})=\sigma^{2}(\Sigma_{0}-\Delta \Sigma^{-1}\Delta^{\prime }) \end{array}$$

The GLS criterion can be employed to jointly estimate all the parameters associated to the model and can be expressed as:

$$\begin{array}{c} \underset{K_{x},K_{\beta },b,\phi }{min}GLS=\underset{K_{x},K_{\beta },b,\phi }{min}{\left(y-Zb\right)}^{\prime }\Sigma {\left(\phi \right)}^{-1}(y-Zb), \end{array}$$

where the parameters *K*_*x*_ and *K*_*β*_ related to the basis for X and *β* are typically chosen *a priori* taking into account, for instance, the quality of the data and its representation on the discretization grid or other considerations related to the data-generating process (smoothness, physical restrictions, interpretability,…). The direct minimization of GLS usually cannot be affordable even though we only consider the parameters **b** and *ϕ*. The generalized cross-validation (GCV) criterion has been widely used to this end despite not being the right criterion for dependent errors. We use the generalized correlated cross-validation (GCCV) as a better alternative. This suggested criterion is an extension to GCV within the context of correlated errors proposed by Carmack et al. [40]. It is defined as follows:

$$\begin{array}{c} GCCV\left(K_{x},K_{\beta },b,\phi \right)=\frac{\sum_{i=1}^{n}{\left(y_{i}-{\hat{y}}_{i,b}\right)}^{2}}{{\left(1-\frac{tr(G)}{n}\right)}^{2}} \end{array}$$

where **G** = 2**H** **Σ**(*ϕ*) − **H** **Σ**(*ϕ*)**H**^**′**^ takes into account the effect of the dependence, the trace of **G** is an estimation of the degrees of freedom consumed by the model and **H** is the hat matrix. The important advantage of this criterion is that it is rather easy to compute because it avoids the need to compute the inverse of the matrix Σ. Even so, the complexity of the GLS criterion depends on the structure of Σ and it could sometimes be hard either to minimize or computationally expensive.

We implement the function fregre.gls (and predict.fregre.gls) that estimates (and predicts) the functional regression model with correlated errors, see S1 Appendix. The fregre.gls function calls the gls function of nlme package. Therefore, the correlation structures allowed are those programmed by the original authors of the package [41].

#### Estimation of functional iterative GLS (iGLS)

The above GLS criterion is employed to jointly estimate all the parameters associated with the model: *K*_*x*_, *K*_*β*_, **b** and *ϕ*. One possibility to alleviate the computational burden is to separate the estimation of the dependence structure (*ϕ*) from the parameters associated to the regression (*K*_*x*_, *K*_*β*_, **b**) in an iterative way (called iGLS) as it is done in multivariate regression. The iGLS proven to be equivalent to classical GLS (see, for instance, [42]). Additionally, the method could consider more flexible dependence models (for instance, selecting the order of an AR instead of fixing it in advance) that avoid the risk of misspecification in the dependence structure. We extend this procedure to the functional regression in the following iterative procedure (called functional iGLS):

1. Begin with a preliminary estimation of $\hat{\phi }=\phi_{0}$ (for instance, *ϕ*_0_ = 0). Compute $\hat{W}$.
2. Estimate $b_{\Sigma }={\left(Z^{\prime }\hat{W}Z\right)}^{-1}Z^{\prime }\hat{W}y$
3. Based on the residuals, $\hat{e}=(y-Zb_{\Sigma })$, update $\hat{\phi }=\rho (\hat{e})$ (and consequently, $\hat{W}$) where *ρ* is subject to the dependence structure chosen.
4. Repeat steps 2 and 3 until convergence (small changes in **b**_Σ_ and/or $\hat{\phi }$)

The estimation of functional $\hat{\beta (t)}$ by **b**_Σ_ is done in step (2), and separated from the estimation of dependence structure *ρ* in step (3). This allows for the flexibility of including any type of dependence structures designed by the user (for instance, using particular restrictions) that are typically not included in the usual packages (like nlme).

We implement, the function fregre.igls (and predict.fregre.igls) that estimates (and predicts) the functional regression model with correlated errors using the iterative scheme (iGLS). We have developed the following two simple structures for Σ in fda.usc package [45] for fit serial dependence structure:

- In iGLS-AR(*p*) scheme, the procedure automatically fits the autoregressive order *p* in each iteration of the errors defined by the equation $\epsilon_{i}=\sum_{j=1}^{p}\phi_{j}\epsilon_{i-j}+\varepsilon_{i}$ where *ε*_*i*_ ∼ *N*(0, *σ*^2^).
- In iGLS-ARMA(*p*,*q*) scheme, the user must specify the parameters *p* and *q* of the autoregressive–moving–average (ARMA(*p*,*q*)) model, which fits the serial error dependence defined by equation: $\epsilon_{i}=\sum_{j=1}^{p}\phi_{j}\epsilon_{i-j}+\sum_{j=1}^{q}\theta_{j}\epsilon_{i-j}+\varepsilon_{i}$ where *ε*_*i*_ ∼ *N*(0, *σ*^2^). This structure is provided by the nlme package but it has a restriction: all parameters of the AR side must be lower than one in absolute value. This rule clearly does not include all the possible stationary models of that order (this is only true for ARMA(1,*q*)).

For these structures, we have used the basic functions ar and arima of the stats package to fit the AR(*p*) and ARMA(*p*,*q*) models, respectively. The users can define their own functions or use other well-known functions that exactly fit the situation at hand.

## Simulation

We have used two functional linear models (FLM) included in [17] to compare the effect of the temporal dependence. Specifically, we have generated *n*_*B*_ = 1000 replicas of size *n* = 100 from the FLM model y=〈X,β〉+ϵ, being X a Wiener process observed in a grid of length *M* = 100 in the interval [0, 1] and *ϵ* an AR(1) process with autoregressive parameter *ϕ* and variance Var(ϵ)=snrVar(〈X,β〉), where *snr* is the signal to noise ratio. For each sample, ten future values, denoted by (*y*_*n*+*h*_, *h* = 1, …, 10), were generated to check the predictive ability of the proposal.

The two models differ only in the *β* parameter that are respectively:

1. *β*(*t*) = 2 sin(0.5*πt*) + 4 sin(1.5*πt*) + 5 sin(2.5*πt*), *t* ∈ [0, 1],
2. *β*(*t*) = log(15*t*^2^ + 10) + cos(4*πt*), *t* ∈ [0, 1].

The scenario (a) corresponds to a *β* parameter which has an exact representation respect to the first three theoretical principal components of the Wiener process. On the contrary, the *β* parameter for scenario (b) cannot be well represented using a small number of theoretical principal components. In both scenarios, we have used two types of basis for representing X and *β*: the empirical principal components basis derived from the sample (FPC) and the cubic B–splines (BSP) at equispaced knots in [0, 1]. The same basis was employed for both representations i.e. in this case Ψ = *φ* and *K*_*x*_ = *K*_*β*_. The optimal number of components (*K*_*β*_) was selected using the GCCV criterion in the range 1–8 for FPC and 5–11 for BSP.

For sake of simplicity, we only show here the results for model (a). The results for model (b) can be revised in the S2 Appendix of the Supporting information.

Tables 1 to 4 summarize the results for the first model (a) to show, respectively, the average number of selected components chosen using GCCV criterion, the mean square error (MSE) for estimation of *β*, the MSE for estimation of *ϕ* and the mean square prediction errors (MSPE) for horizons 1, 5 and 10. In these results, LM denotes the estimation through a classical functional linear model whereas GLS and iGLS corresponds, respectively, to the functional GLS and functional iGLS methods (shown in Methodology section for AR(1) dependent errors).

**Table 1:** Average of number of basis elements selected by GCCV criterion in *n*_*B*_ = 1000 replicas in model (a).

	PC			BSP		
*snr*	*ϕ* = 0	*ϕ* = 0.5	*ϕ* = 0.9	*ϕ* = 0	*ϕ* = 0.5	*ϕ* = 0.9
0.05	3.3	3.3	3.3	6.4	6.5	6.6
0.10	3.2	3.2	3.3	5.9	6.0	6.1

$$\begin{array}{c} E({\left||\beta -\hat{\beta }|\right|}^{2}) \end{array}$$

**Table 2:** Mean square error of *β* parameter. Model (a), *n*_*B*_ = 1000.

		PC			BSP		
*snr*	Model	*ϕ* = 0	*ϕ* = 0.5	*ϕ* = 0.9	*ϕ* = 0	*ϕ* = 0.5	*ϕ* = 0.9
0.05	LM	0.457	0.442	0.443	0.996	1.014	0.965
0.05	GLS-AR(1)	0.457	0.421	0.400	0.997	0.813	0.493
0.05	iGLS-AR(1)	0.457	0.421	0.400	0.997	0.813	0.493
0.05	iGLS-AR(p)	0.457	0.421	0.400	1.001	0.816	0.494
0.10	LM	0.501	0.502	0.503	1.243	1.261	1.218
0.10	GLS-AR(1)	0.501	0.471	0.437	1.244	1.031	0.661
0.10	iGLS-AR(1)	0.501	0.471	0.437	1.244	1.031	0.661
0.10	iGLS-AR(1)	0.501	0.471	0.437	1.247	1.032	0.662

$$\begin{array}{c} E({\left(\phi -\hat{\phi }\right)}^{2}) \end{array}$$

**Table 3:** Mean square error of *ϕ* parameter. Model (a), *n*_*B*_ = 1000.

		PC			BSP		
*snr*	Model	*ϕ* = 0	*ϕ* = 0.5	*ϕ* = 0.9	*ϕ* = 0	*ϕ* = 0.5	*ϕ* = 0.9
0.05	GLS-AR(1)	0.004	0.003	0.001	0.004	0.003	0.001
0.05	iGLS-AR(1)	0.004	0.003	0.002	0.004	0.003	0.001
0.10	GLS-AR(1)	0.004	0.003	0.001	0.004	0.003	0.001
0.10	iGLS-AR(1)	0.004	0.003	0.001	0.004	0.003	0.001

$$\begin{array}{c} MSPE=\frac{1}{n_{B}}\sum_{b=1}^{n_{B}}{\left(y_{n+h}^{b}-{\hat{y}}_{n+h}^{b}\right)}^{2} \end{array}$$

**Table 4:** Mean square prediction errors for lags *h* = 1, 5 and 10. Model (a), *n*_*B*_ = 1000.

			AR(1)								
			*ϕ* = 0			*ϕ* = 0.5			*ϕ* = 0.9		
*snr*	Model	Basis	h = 1	h = 5	h = 10	h = 1	h = 5	h = 10	h = 1	h = 5	h = 10
0.05	LM	PC	0.070	0.068	0.065	0.070	0.077	0.068	0.070	0.070	0.070
0.05	GLS-AR(1)	PC	0.070	0.068	0.065	0.054	0.077	0.068	0.015	0.047	0.061
0.05	iGLS-AR(1)	PC	0.070	0.068	0.065	0.054	0.077	0.068	0.015	0.047	0.061
0.05	iGLS-AR(*p*)	PC	0.070	0.068	0.065	0.055	0.076	0.068	0.015	0.047	0.062
0.05	LM	BSP	0.071	0.069	0.066	0.071	0.077	0.068	0.071	0.072	0.070
0.05	GLS-AR(1)	BSP	0.071	0.069	0.066	0.054	0.076	0.068	0.015	0.046	0.060
0.05	iGLS-AR(1)	BSP	0.071	0.069	0.066	0.054	0.076	0.068	0.015	0.046	0.060
0.05	iGLS-AR(*p*)	BSP	0.072	0.069	0.066	0.055	0.076	0.068	0.015	0.047	0.061
0.10	LM	PC	0.137	0.152	0.126	0.150	0.137	0.141	0.136	0.136	0.153
0.10	GLS-AR(1)	PC	0.137	0.152	0.126	0.114	0.137	0.140	0.030	0.094	0.136
0.10	iGLS-AR(1)	PC	0.137	0.152	0.126	0.114	0.137	0.140	0.030	0.093	0.136
0.10	iGLS-AR(*p*)	PC	0.138	0.152	0.126	0.115	0.138	0.140	0.030	0.094	0.136
0.10	LM	BSP	0.138	0.153	0.130	0.150	0.137	0.143	0.140	0.137	0.155
0.10	GLS-AR(1)	BSP	0.139	0.153	0.130	0.114	0.137	0.140	0.029	0.094	0.136
0.10	iGLS-AR(1)	BSP	0.139	0.153	0.130	0.114	0.137	0.140	0.029	0.093	0.135
0.10	iGLS-AR(*p*)	BSP	0.140	0.153	0.130	0.115	0.137	0.140	0.030	0.094	0.136

Table 1 shows an average number of FPC selected components between 3 and 4 with a slight tendency to lower values as the *snr* grows. The average number of B–splines basis was between 6 and 7 although in this case we do not have a theoretical quantity to compare with. It seems that there are no trends with respect to the *ϕ* values. Table 2 clearly shows the advantage of the PC estimator over the B–splines because the estimation error using B–splines typically doubles the error using PCs. In this table, we can also see the improved estimates of the GLS and iGLS method over the LM, especially when *ϕ* grows. The same equivalence is shown in Table 3 for the mean square error (MSE) of the *ϕ* parameter, which shows better results as the dependence grows. Finally, Table 4 shows the mean square prediction errors (MSPE) for different lags showing a clear improvement of GLS procedures, specially for large *ϕ* and shorter lags. With respect to the prediction ability between PC or B–splines, the results show that both methods are almost equivalent with minor differences along the table.

Table 5 summarizes the results of the Model (a) but replaces the AR(1) by an AR(2) error process using the FPC estimation (the results with BSP are similar). In all these models, the minimum square prediction error is achieved with model iGLS-AR(2) in which an AR(2) is estimated in each iteration of the algorithm. This is followed very closely by model iGLS-AR(*p*), estimating an automatic choice of *p* at each iteration.

$$\begin{array}{c} MSPE=\frac{1}{n_{B}}\sum_{b=1}^{n_{B}}{\left(y_{n+h}^{b}-{\hat{y}}_{n+h}^{b}\right)}^{2} \end{array}$$

**Table 5:** Mean square prediction errors for different lags *h* = 1, 5, 10. Estimation of Model (a) using PC with an AR(2) error process.

		AR(p = 2)								
		(*ϕ*_1_ = 0.5, *ϕ*_2_ = 0.45)			(*ϕ*_1_ = 1.4, *ϕ*_2_ = −0.45)			(*ϕ*_1_ = 1.5, *ϕ*_2_ = −0.75)		
*snr*	Model	*h* = 1	*h* = 5	*h* = 10	*h* = 1	*h* = 5	*h* = 10	*h* = 1	*h* = 5	*h* = 10
0.05	LM	0.0707	0.0693	0.0725	0.0643	0.0628	0.0667	0.0657	0.0688	0.0737
0.05	GLS-AR(1)	0.0144	0.0304	0.0490	0.0154	0.0493	0.0626	0.0191	0.1072	0.0714
0.05	iGLS-AR(1)	0.0144	0.0309	0.0497	0.0154	0.0483	0.0600	0.0191	0.1055	0.0712
0.05	iGLS-AR(2)	0.0109	0.0250	0.0415	0.0050	0.0358	0.0551	0.0092	0.0572	0.0682
0.05	iGLS-AR(p)	0.0115	0.0256	0.0424	0.0052	0.0364	0.0554	0.0093	0.0569	0.0682

The first AR(2) process, (*ϕ*_1_ = 0.5, *ϕ*_2_ = 0.45), is roughly like an AR(1) process with *ϕ* ≈ 0.95. This can explain why the results of the iGLS-AR(1) model are so close to the optimum estimated by the iGLS-AR(2). The second AR(2) process, (*ϕ*_1_ = 1.4, *ϕ*_2_ = −0.45), was selected to assess the misspecification error. Although the use of an AR(1) process in the GLS and iGLS models improves the LM model, these results are far from the best using an AR(2) specification. The autocorrelation function of the AR(2) process shows a periodicity pattern that cannot be approximated by an AR(1) process. Finally, the third AR(2), (*ϕ*_1_ = 1.5, *ϕ*_2_ = −0.75), shows the effect of the misspecification in a later horizon *h* = 5, making the results at that horizon for an AR(1) specification even worse than the LM model. Again, this is motivated by the periodicity pattern of the AR(2) due to the negative sign of *ϕ*_2_. In all cases, the specification iGLS–AR(*p*) is rather close to the optimum. However, the important advantage is that it avoids a closed specification form of the dependence structure. Finally, the GLS-AR(2) scenario was not considered in this table because the gls function of nlme package does not allow the estimation of any parameter of an AR(2) greater than 1 in absolute value. This is an empirical rule in the package that avoids the use of non stationary processes although, in this case, the three AR(2) specifications are clearly stationary, but only the first specification can be estimated using the gls function.

## An application to Galician flu prediction rate

Galicia is a region of 29, 574 km^2^ located in Northwest Spain with a population of 2.8 million people. We analyzed the weekly incidence of reported cases of influenza in Galicia between 2001 and 2011 for each of the 53 Galician counties:

$$\begin{array}{c} {\text{Rate}}_{n,s}=\log\left(cases_{n,s}\times 100000/pop_{n,s}\right) \end{array}$$

for county *s* and week *n*. The population (*pop*) was obtained from the Statistical Institute of Galicia (IGE, http://www.ige.eu) and the number of influenza cases (*cases*) from the Health Service of Galicia (www.sergas.es).

The influenza season in Galicia usually begins in week 40 and ends in week 20 of the following year. The goal is to predict the incidence of influenza for the following two weeks (*n* + 1 and *n* + 2) for each of the *s* regions with the available information:

- Rate_*n,s*_(*w*): Weekly influenza rate for last 13 weeks, *w* ∈ [*n* − 12, *n*].
- Temp_*n,s*_(*t*): Daily temperature in Celsius degrees (°C) for last 14 days, *t* ∈ [*n* − *i*/7, *n*], for *i* = 14, …, 1.
- Dushoff et al. [7] defined cold as the number of degrees below a threshold temperature: Temp.th_*n,s*_ = min(Temp_*n,s*_ − thres, 0) with thres = 10°*C*. The functional variable is defined as: Temp.th_*n,s*_(t) with *t* ∈ [*n* − *i*/7, *n*], for *i* = 14, …, 1.
- SR_*n,s*_(*t*): Daily solar radiation (*W*/*m*^2^) for the last 14 days, *t* ∈ [*n* − *i*/7, *n*], for *i* = 14, …, 1.
- Hum_*n,s*_(*t*): Relative humidity for the last 14 days: *t* ∈ [*n* − *i*/7, *n*], for *i* = 14, …, 1.

**Fig 1:**
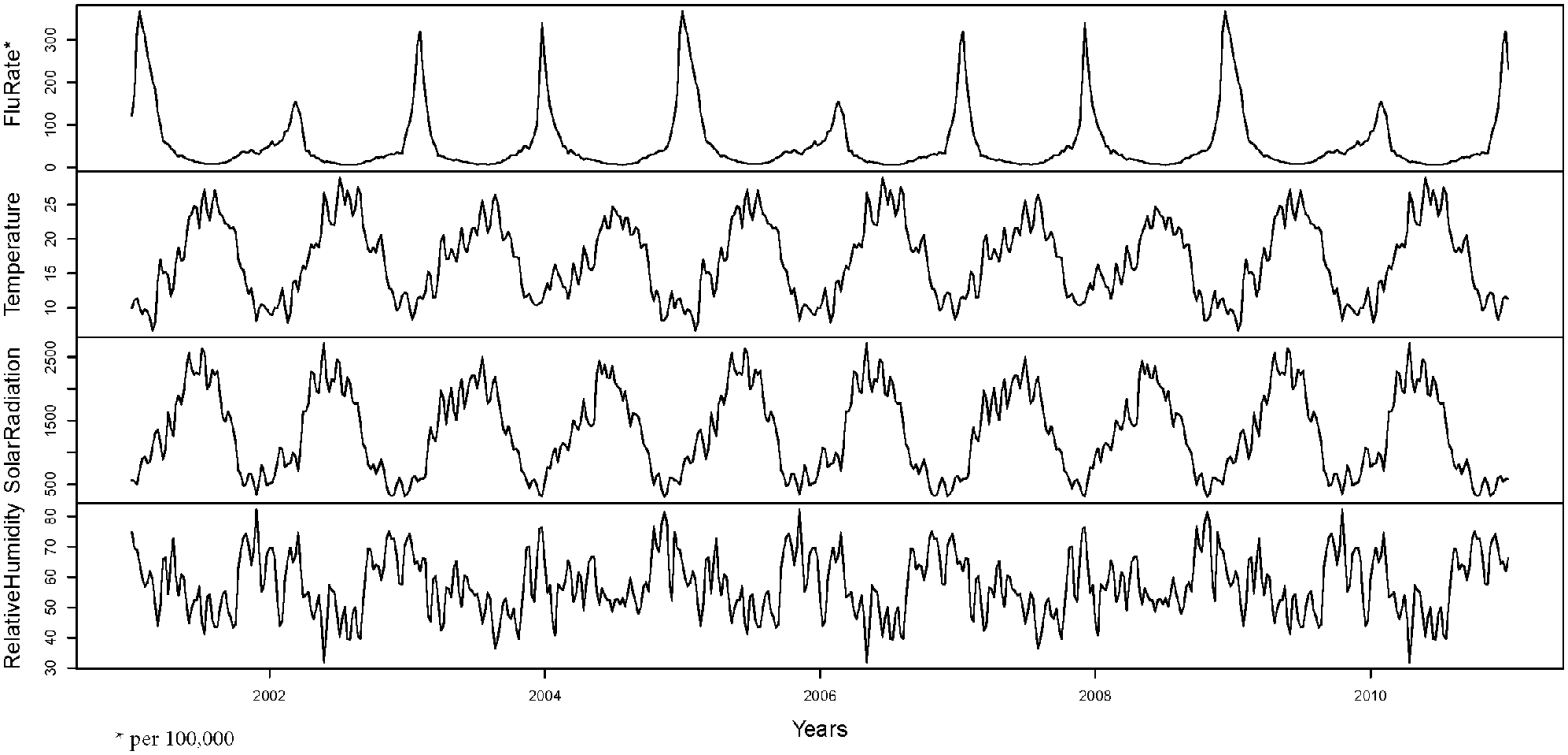
Influenza rate and meteorological covariates. From top to bottomml: Overall weekly influenza rate, and daily average temperature, solar radiation and relative humidity in the Galician region during the period.

For representing the above functional covariates, a B–spline basis of five components was used in all cases (based on the previous experience of the authors with this type of data). The prediction for the overall influenza rate is constructed by appropriately aggregating the predictions of the *s* regions that are made independently, i.e. the estimation of *β* and *ϕ* are made only with the data of that county. Fig 1 shows the overall influenza rate that normally grows in the late autumn and reaches a peak at the beginning of the calendar year. These plots clearly show the large difference between reported influenza cases in winter and summer. The influenza rate for each county shows a similar pattern but with small differences in the peak epidemic period. We downloaded meteorological data from the regional Weather Service of Galicia (http://www.meteogalicia.es/). S1 Appendix describes the supplementary material (functions, libraries, source data and code) and S1 File contains the code and dataset used in this study.

### Variable selection using distance correlation measure

Distance correlation R is a measure of dependence between random vectors introduced by Székely et al. [43]. The distance correlation satisfies 0≤R(X,Y)≤1 and its interpretation is similar to the squared Pearson’s correlation. However, the advantages of distance correlation over the Pearson correlation is that it defines R(X,Y) in arbitrary finite dimensions of *X* and *Y* and R characterises independence, i.e. R(X,Y)=0⇔X,Y are independent. Recently, Lyons [44] provided conditions for the application of the distance correlation to functional spaces. So, this measure seems to be a good indicator of the correlations between functional and multivariate variables that may be useful for designing a functional linear model (for instance, avoiding variates with high collinearity). The empirical distance correlation $R_{n,s}\left(X,Y\right)$ can be easily computed as

$$\begin{array}{c} R_{n,s}\left(X,Y\right)=\sqrt{\frac{V_{n,s}^{2}\left(X,Y\right)}{\sqrt{V_{n,s}^{2}\left(X\right)V_{n,s}^{2}\left(Y\right)}}}. \end{array}$$

where $V_{n,s}\left(X,Y\right)$ is the empirical distance covariance defined by

$$\begin{array}{c} V_{n,s}^{2}\left(X,Y\right)=\frac{1}{n^{2}}\sum_{k,\;l=1}^{n}A_{kl}B_{kl} \end{array}$$

where $A_{kl}=a_{kl}-{\bar{a}}_{k.}-{\bar{a}}_{.l}+{\bar{a}}_{..}$ and $B_{kl}=b_{kl}-{\bar{b}}_{k.}-{\bar{b}}_{.l}+{\bar{b}}_{..}.$ with *a*_*kl*_ = ‖*X*_*k*_ − *X*_*l*_‖, *b*_*kl*_ = ‖*Y*_*k*_ − *Y*_*l*_‖, *k*, *l* = 1, …, *n*, and the subscript. denotes that the mean is computed for the index that it replaces. Similarly, $V_{n,s}\left(X\right)$ is the non-negative number defined by $V_{n,s}^{2}\left(X\right)=V_{n,s}^{2}\left(X,X\right)=\frac{1}{n^{2}}\sum_{k,\;l=1}^{n}A_{kl}^{2}$.

The distance correlation R was used to select the information relevant to the prediction of influenza rate not only with respect to the response but also among the possible covariates to avoid collinearities. The results are shown in Table 6. Relative humidity, Hum_*n,s*_(*t*), has the lowest correlation with the influenza rate {Rate_*n*+1,*s*_, Rate_*n*+2,*s*_} and therefore, it seems that its contribution to the response is negligible (a model with Hum_*n,s*_(*t*) never improves one without the variate). Besides, the distance correlation values are useful for designing models avoiding closely related covariates (for instance, Temp_*n,s*_(*t*) and Temp.th_*n,s*_(*t*) share the same information). With these considerations, the number of possible different models to be tested is quite reduced.

**Table 6:** Distance correlation R between the response at week *n* + 1 and *n* + 2 and functional covariates at week *n*.

R	Rate_*n,s*_(*w*)	Temp_*n,s*_(*t*)	Temp.th_*n,s*_(*t*)	SR_*n,s*_(*t*)	Hum_*n,s*_(*t*)	Rate_*n*+1,*s*_	Rate_*n*+2,*s*_
Rate_*n,s*_(*w*)	1.00	0.56	0.48	0.43	0.26	0.69	0.64
Temp_*n,s*_(*t*)	0.56	1.00	0.90	0.78	0.54	0.52	0.50
Temp.th_*n,s*_(*t*)	0.48	0.90	1.00	0.73	0.44	0.46	0.45
SR_*n,s*_(*t*)	0.43	0.78	0.73	1.00	0.72	0.52	0.51
Hum_*n,s*_(*t*)	0.26	0.54	0.44	0.72	1.00	0.31	0.30

### Prediction using temporal dependence structure

A rolling analysis was employed to compare the models in a predictive scenario. Initially, a series of length *j* = 1, …, *n* = 150 weeks in *s* = 53 counties is used to predict the influenza rate in the next two weeks, *n* + 1 and *n* + 2. The rolling is then performed along the epidemic periods (*J* = 28 weeks, from week 40 to week 15 next year) by computing the mean square predictive error:

$$\begin{array}{c} MSPE=\frac{1}{J}\sum_{j=n+1}^{n+J}\sum_{r=1}^{s}w_{r}{\left({Rate}_{j,r}-{\hat{Rate}}_{j,r}\right)}^{2} \end{array}$$

where *w*_*r*_ is the weight (in terms of *pop*) for county *r*. For ease of simplicity, the GLS setting is only considered with an AR(1) specification of the dependence structure, whereas the iGLS is combined with an AR(1), AR(2) and AR(*p*).

Table 7 summarises the MSPE for the influenza season. The best result for each set of covariates is shaded in light gray and the overall winner for each horizon is in bold font. In the models with the predictor Rate_*n,s*_(*w*) (rows (a), (e), (f) and (g)) the gain, in terms of MSPE, of the functional GLS models (GLS–AR(1), iGLS–AR(1), iGLS–AR(2) and iGLS–AR(*p*)) is relatively small with respect to functional LM models because the Rate_*n,s*_(*w*) partly accounts for the temporal dependence. Furthermore, in some sense, the inclusion of the predictor Rate_*n,s*_(*w*) in the model is akin to the estimation of the dependence structure. The models without influenza rate (rows (b), (c), (d) and (h)) begin with a worse result in the LM setting, but their results become competitive (or even become the best ones) with the inclusion of the serial dependence. The difference between the GLS or iGLS setting is that the latter allows more flexibility, not only defining a different dependence structure in each county, but also in the estimation of that dependence. This is particularly useful when the forecast horizon increases. The GLS setting must fix the order of the AR in advance and, when the number of regions is high, it is a tough assumption to consider the order of the serial dependence model fixed for all of them. For *n* + 1 the best models are (b) and (c) with GLS–AR(1) and iGLS–AR(1) specifications, using the curve of temperature of last 14 days as the predictor and a simple AR(1) structure for the adjustment of the residuals. The best autoregressive model estimated by the iGLS–AR(*p*) model has been, in most cases, of order 1. For *n* + 2, in some regions, an AR(1) or AR(2) model may be insufficient; the best result is achieved with the iGLS–AR(*p*) procedure, which presents greater flexibility in estimating the different *p* order for each county.

$$\begin{array}{c} MSPE=\frac{1}{J}\sum_{j=n+1}^{n+J}\sum_{r=1}^{s}w_{r}{\left({Rate}_{j,r}-{\hat{Rate}}_{j,r}\right)}^{2} \end{array}$$

**Table 7:** Mean square predictive error for influenza period using the rolling procedure.

	*n* + 1				
Covariates	LM	GLS-AR(1)	iGLS-AR(1)	iGLS-AR(2)	iGLS-AR(*p*)
(a) Rate_*n*, *s*_(*w*)	0.510	0.404	0.404	0.405	0.402
(b) Temp_*n*, *s*_(*t*)	1.177	**0.362**	**0.362**	0.364	0.379
(c) Temp.th_*n*, *s*_(*t*)	2.530	0.391	0.391	0.402	0.418
(d) SR_*n*, *s*_(*t*)	1.290	0.381	0.381	0.394	0.407
(e) Rate_*n*, *s*_(*w*), Temp_*n*, *s*_(*t*)	0.487	0.404	0.404	0.392	0.390
(f) Rate_*n*, *s*_(*w*), Temp.th_*n*, *s*_(*t*)	0.538	0.362	0.448	0.441	0.437
(g) Rate_*n*, *s*_(*w*), SR_*n*, *s*_(*t*)	0.505	0.418	0.418	0.409	0.404
(h) Temp_*n*, *s*_(*t*), SR_*n*, *s*_(*t*)	1.163	0.384	0.384	0.389	0.402
	*n* + 2				
Covariates	LM	GLS-AR(1)	iGLS-AR(1)	iGLS-AR(2)	iGLS-AR(*p*)
(a) Rate_*n*, *s*_(*w*)	0.931	0.903	0.901	0.849	0.809
(b) Temp_*n*, *s*_(*t*)	1.250	0.785	0.764	0.760	**0.712**
(c) Temp.th_*n*, *s*_(*t*)	1.954	0.841	0.830	0.828	0.792
(d) SR_*n*, *s*_(*t*)	1.272	0.821	0.823	0.834	0.795
(e) Rate_*n*, *s*_(*w*), Temp_*n*, *s*_(*t*)	0.883	0.879	0.814	0.810	0.764
(f) Rate_*n*, *s*_(*w*), Temp.th_*n*, *s*_(*t*)	0.911	0.785	0.874	0.856	0.800
(g) Rate_*n*, *s*_(*w*), SR_*n*, *s*_(*t*)	0.951	0.939	0.880	0.877	0.855
(h) Temp_*n*, *s*_(*t*), SR_*n*, *s*_(*t*)	1.273	0.796	0.784	0.783	0.746

Models (b) and (c), with GLS setting, present slight differences. Of course, it seems better to use the temperature than to only use the threshold respect to a level. Yet the differences between these two models suggest that the evolution of temperatures when it is cold is crucial to explaining the influenza rate. Model (h) makes no improvement on the results of models (b) and (c) in terms of MSPE. In fact, it worsens them; this is probably due to collinearity among Temp_*n,s*_(*t*) and SR_*n,s*_(*t*). Concerning models (b), (c) and (d), the first two are preferable because they are easier to apply and interpret. Besides, in model (d) the measures of solar radiation usually depend on specialised devices, whereas the covariates related to temperature are readily available using standard (and cheaper) equipment. Finally, for short horizons, it seems unnecessary to specify high order autoregressive models, even though the improvement can be about 5% for larger lags.

**Fig 2:**
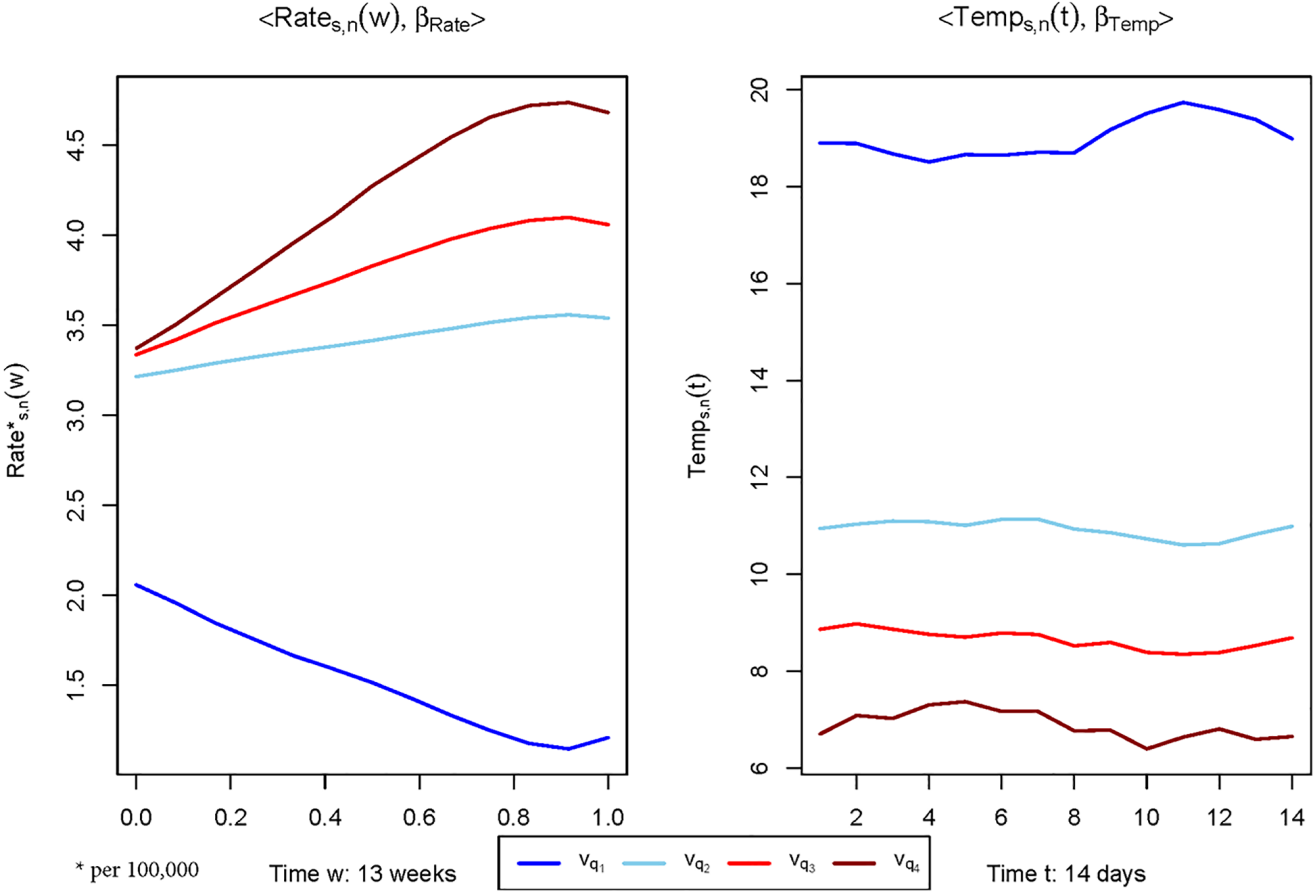
Shape of the covariates with respect to their contribution in the model. Shape of rate curves (on left) and temperature threshold curves (on right) categorized by their projection value $v_{X}=\langle X,\hat{\beta }\rangle$. The groups are constructed as a function of the quantile of *v*_*X*_ (*q*(*v*_*X*_)): *q*(*v*_*X*_) ∈ [0, .25] (dark blue line), *q*(*v*_*X*_) ∈ (0.25, 0.50] (blue line), *q*(*v*_*X*_) ∈ (0.50, 0.75] (red line) and *q*(*v*_*X*_) ∈ (0.75, 1] (dark red line).

Indeed, it is possible to interpret the $\hat{\beta }$ parameter associated with models. To this end, we have computed for models (a) and (b), the quantities $v_{i}=\langle X_{i},\hat{\beta }\rangle$, which are the contribution of every curve to the influenza rate. So, if we classify the curves in groups according to these values and average them, we can see the pattern of the curves that have the most (or least) influence with respect to the incidence rate. This is done in Fig 2, which shows the pattern of curves that most contributed to increasing (in red scale) and decreasing (in blue scale) the influenza rate. In particular, we have split the data with respect to the quartiles of *v*_*i*_ and assigned (from bottom to top) the following colors: blue, sky blue, red and dark red. This assesses the evaluation of the contribution of these curves in the response. So, as expected, the contribution of an intense increasing pattern of the influenza rate in the last weeks is plotted in dark red (see left panel of Fig 2), which leads to predicting high influenza rates. On the other hand, a decreasing pattern is plotted in dark blue, meaning that this type of pattern corresponds with low influenza rates. The same reasoning can be applied to model (b)(see right panel of Fig 2). Curves of temperature below 7°C are plotted in dark red, meaning that this pattern provides high prediction rates. On the other hand, the curves around 19°C (plotted in dark blue) lead to almost zero influenza rates. The dark red line corresponds to the pattern of the curves that most contribute to increasing the estimated incidence rate. In the week *w* = 1 begins $v_{q_{4}}\approx 3.3$ that, if we undo the logarithmic transformation represents an incipient incidence of 27.1 cases per 100, 000 population and goes up monotonously until last register (*w* = 13), which takes the value $v_{q_{4}}\approx 4.6$, implying an increase of 99.5 cases per 100, 000 population.

**Fig 3:**
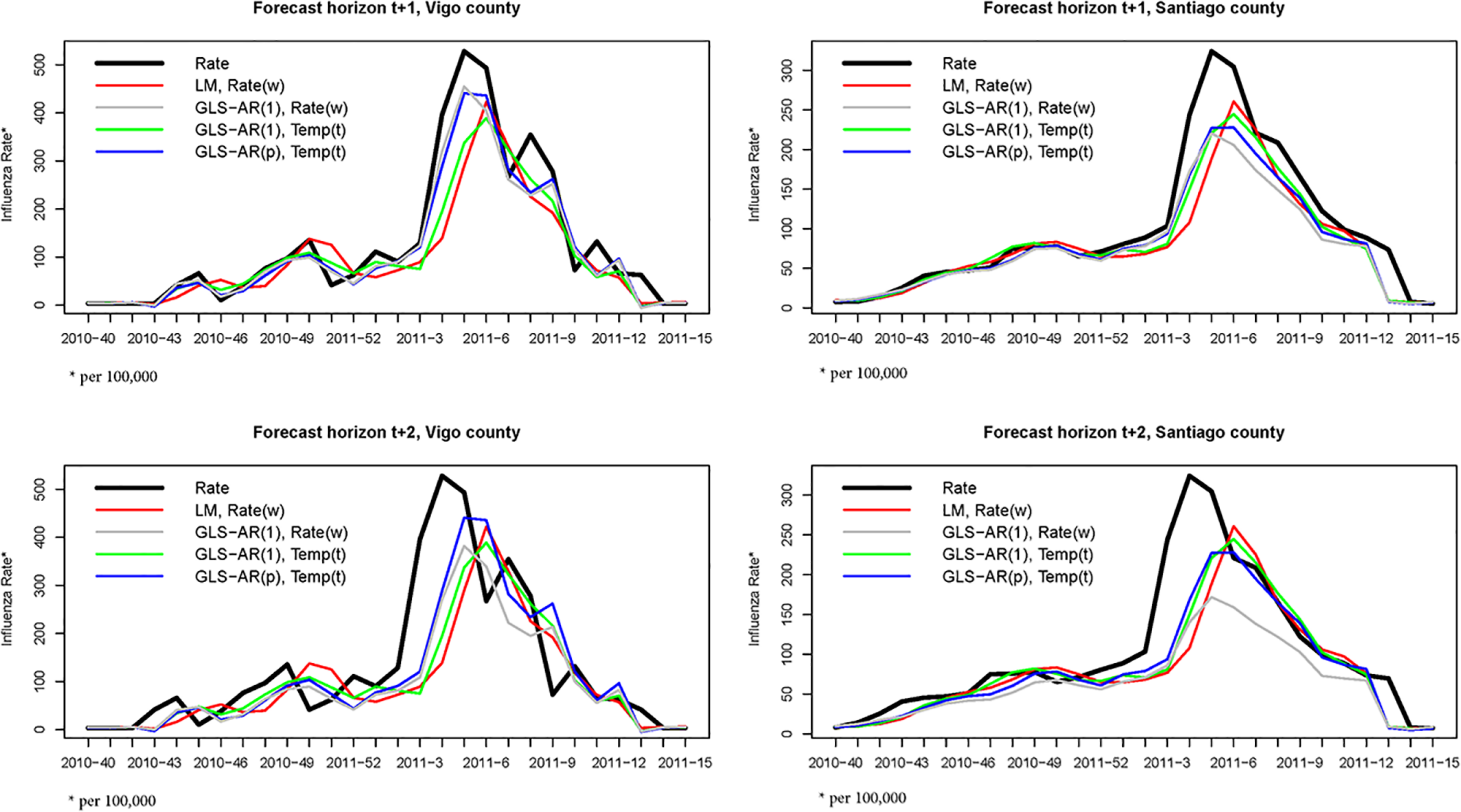
Example of raw influence rate prediction. Prediction of the raw rate (*cases* × 100000/*pop*) for two counties (Vigo and Santiago) in Galicia using four models: LM, *Rate*(*w*); GLS–AR(1), *Rate*(*w*); GLS-AR (1), *Temp*(*t*) and GLS-AR(p), *Temp*(*t*). In each case, the raw rate is compared with the prediction provided one week before (*t* + 1, first row) and two weeks before (*t* + 2, second row). The counties are separated by columns.

Finally, as an illustration in Fig 3 the prediction of the raw rate (*cases* × 100000/*pop*) during the 2010–11 flu epidemic season is provided for two counties (Vigo and Santiago) as a result of reversing the log transform of the response in the preceding models. In both counties, the peak is achieved at week 2011–5 (first week of February). The two considered horizons (*t* + 1 and *t* + 2) are shown by rows. In each case, the raw rate is compared with the prediction obtained one or two weeks before with the models LM, *Rate*(*w*); GLS–AR(1), *Rate*(*w*); GLS–AR(1), *Temp*(*t*) and GLS–AR(*p*), *Temp*(*t*). Focusing on *t* + 1, the comparison among the two dependence structures (AR(1) and AR(*p*), lines green and blue, respectively) associated with *Temp*(*t*) shows a big difference for Vigo but no for Santiago. This suggests that for Santiago an AR(1) is enough whereas for Vigo it seems more adequate a general AR(*p*) specification. Respect to the models including the *Rate*(*t*) (lines red and gray), the model using GLS reacts faster than the LM model providing better predictions of the peak. Predictions for medium or low intensities (below 125) are quite similar. For *t* + 2, no clear patterns are shown, although the specification GLS–AR(*p*) seems to do slightly better.

## Conclusion

This paper extends the GLS model from a multivariate to a functional framework: it thereby allows us to estimate functional regression models with temporal or spatial covariance errors structure in a simple way. It proposes an iterative version of the GLS estimator, that can help to model very complicated dependence structures. This procedure (called iGLS) is much simpler than GLS in terms of the optimization function to be accomplished but, of course, it may take longer due to the iterations. However, iGLS may be the only option when the sample size or the dimension of the parameter increases and the joint optimization performed by GLS is not affordable (in terms of complexity or memory consumption).

A simulation study shows that the GLS estimators improve the classical approach because they provide better estimations of the parameters associated with the regression model and extremely good results from the predictive point of view, specially for short lags.

The GLS procedures have been applied to the prediction of the influenza rate using readily available functional variables. These kinds of models are extremely useful to health managers in allocating resources in advance for an epidemic outbreak. The estimation of the dependence allows that simpler models can achieve good results maintaining nice interpretations of the model. In particular, the simple model (b) that only uses the easy-to-measure variate Temp_*n,s*_(*t*), shows that influenza may increase due to a cold wave with daily temperatures around 7°C for two weeks which is consistent with much of the literature on influenza. Also, the models show that the estimated temporal dependence of the influenza virus is strong and stable over time.

In our examples, we estimated the error structure with simple AR(*p*) models (mostly AR(1) or AR(2)) obtaining a good fit for time dependence. We also tried other ARMA models and obtained similar results. Our method can additionally be used to explore more complex dependence structures like heterogeneous covariances by counties or even spatio–temporal modelling. The iGLS procedure allows for more simplicity and flexibility in the estimation of the dependence structure at the cost of a light heavier computational work. Furthermore, in particular in the example provided, the iGLS allows us to specify a general dependence structure that can be adapted for every county rather than considering the same model for all counties or designing, by hand, the best structure for each county.

## Supporting information

S1 Appendix Complete description of functions, libraries, source data and code used along the paper. (PDF)

S2 Appendix Extended simulation. Simulations results for model (b).
(PDF)

S1 File Supplemental code and data zip file. File containing the code and dataset used along the paper.
(ZIP)
