# Supplementary material for: Predicting seasonal influenza transmission using functional regression models with temporal dependence
Source: PLoS One. 2018 Apr 25;13(4):e0194250. doi: 10.1371/journal.pone.0194250 (PMC5918942; doi:10.1371/journal.pone.0194250)
Supplement: S1 Appendix — (PDF) [file pone.0194250.s001.pdf]

S1 Appendix: Supplemental information, source data and code

This document is an extension of a chapter of a book derived from a congress (IWFOS 2014, Stresa, Italy) [1]. In addition, this work includes new sections that enhancing the simulation study and incorporating a nice application to flu epidemics.

All functions needed for the simulation studies are included in version 1.4 of the `fda.usc` package [2] of software R. The function `fregre.gls` (and `predict.fregre.gls`) estimates (and predicts) the functional regression model with correlated errors, function `fregre.igls` is an iterative version of the previous one, function `dcor.fdist` computes the distance correlation between multivariate and functional objects and function `GCCV.S` computes the GCCV criterion.

`Supplemental Code and Data.zip`: Zip compressed file containing the code for numerical studies (`PLOSsimulation_AR1.R`, `PLOSsimulation_AR2.R`), the real dataset (`influenza.rda`) and the code for generating the plots and tables in the flu prediction example (`influenza.R`).

References

1. Febrero-Bande M, Oviedo de la Fuente M. Functional Regression Models with Temporal and/or Spatial Dependence. In *Contributions in infinite-dimensional statistics and related topics* pp:107–112. Società Editrice Esculapio, 2014.

2. Febrero-Bande M, Oviedo de la Fuente M. Statistical computing in functional data analysis: the R package fda.usc. J Stat Softw. 2012;51(4):1–28.
