# Supplementary material for: Predicting seasonal influenza transmission using functional regression models with temporal dependence
Source: PLoS One. 2018 Apr 25;13(4):e0194250. doi: 10.1371/journal.pone.0194250 (PMC5918942; doi:10.1371/journal.pone.0194250)
Supplement: S2 Appendix — Simulations results for model (b). (PDF) [file pone.0194250.s002.pdf]

S2 Appendix: Simulation (b)

The results for Simulation (b) are summarised in Tables 1, 2, 3 and 4. For the sake of simplicity, the results from the iGLS method are not shown because, as in the previous case, the numbers are almost identical with GLS. In this second model, the estimation of β cannot be done efficiently with the eigenfunctions of the Wiener process and so, the PC method has no a clear advantage over the B-splines. The number of selected components in Table 1 is clearly low for PC and quite unstable for higher values of ϕ. This is also reflected in Table 2 where, especially for $\phi = 0.9$, the estimation error is lower for the B-spline procedure. Note that, the estimation error can be split in two parts: a systematic one due to the lack of representation of β using a particular basis and the approximation one due to the particular estimation of that basis representation with the data at hand. This also affects the estimation of the dependence parameter as it is shown in Table 3 where the mean square errors provided are larger than in the previous model. In any case, the mean square prediction errors in Table 4 are better for the GLS procedures than for the classical one.

	PC			BSP		
snr	$\phi = 0$	$\phi = 0.5$	$\phi = 0.9$	$\phi = 0$	$\phi = 0.5$	$\phi = 0.9$
0.05	2.6	2.6	2.8	5.6	5.6	5.6
0.10	2.0	2.1	2.1	5.5	6.0	5.5

Table 1. Average of number of basis elements selected by GCCV criterion. Model (b), $n_B = 1000$

$$\mathbb{E}\left(\left|\left|\beta - \hat{\beta}\right|\right|^2\right)$$

		PC			BSP		
snr	Model	$\phi = 0$	$\phi = 0.5$	$\phi = 0.9$	$\phi = 0$	$\phi = 0.5$	$\phi = 0.9$
0.05	LM	1.079	1.078	1.058	0.954	0.981	0.950
0.05	GLS–AR(1)	1.080	1.057	1.017	0.956	0.754	0.432
0.05	iGLS–AR(1)	1.080	1.057	1.017	0.956	0.753	0.432
0.05	iGLS–AR(p)	1.080	1.057	1.017	0.962	0.756	0.432
0.10	LM	1.117	1.122	1.119	1.296	1.318	1.235
0.10	GLS–AR(1)	1.118	1.106	1.081	1.295	0.984	0.497
0.10	iGLS–AR(1)	1.118	1.106	1.081	1.295	0.984	0.497
0.10	iGLS–AR(p)	1.118	1.106	1.081	1.300	0.985	0.498

Table 2. Mean square error of β parameter. Model (b), $n_B = 1000$

$$\mathbb{E}\left(\left(\phi - \hat{\phi}\right)^2\right)$$

		PC			BSP		
snr	Model	$\phi = 0$	$\phi = 0.5$	$\phi = 0.9$	$\phi = 0$	$\phi = 0.5$	$\phi = 0.9$
0.05	GLS	0.004	0.004	0.003	0.004	0.003	0.001
0.05	iGLS	0.004	0.004	0.003	0.004	0.003	0.001
0.10	GLS	0.004	0.004	0.002	0.004	0.003	0.001
0.10	iGLS	0.004	0.004	0.003	0.004	0.003	0.001

Table 3. Mean square error of ϕ parameter in a AR(1). Model (b), $n_B = 1000$

$$MSPE = \frac{1}{n_B}\sum_{b=1}^{n_B}\left(y^b_{n+h} - \hat{y}^b_{n+h}\right)^2$$

			AR(p=1)								
			$\phi = 0$			$\phi = 0.5$			$\phi = 0.9$		
snr	Model	Basis	h=1	h=5	h=10	h=1	h=5	h=10	h=1	h=5	h=10
0.05	LM	PC	0.136	0.130	0.126	0.134	0.148	0.127	0.131	0.135	0.131
0.05	GLS–AR(1)	PC	0.136	0.130	0.126	0.104	0.147	0.128	0.032	0.092	0.115
0.05	iGLS–AR(1)	PC	0.136	0.130	0.126	0.104	0.147	0.128	0.032	0.092	0.116
0.05	iGLS–AR(p)	PC	0.136	0.130	0.126	0.104	0.147	0.128	0.032	0.094	0.117
0.05	LM	BSP	0.134	0.128	0.122	0.131	0.143	0.126	0.132	0.132	0.129
0.05	GLS–AR(1)	BSP	0.134	0.128	0.122	0.100	0.142	0.126	0.026	0.086	0.112
0.05	iGLS–AR(1)	BSP	0.134	0.128	0.122	0.100	0.142	0.126	0.026	0.085	0.112
0.05	iGLS–AR(p)	BSP	0.133	0.128	0.122	0.101	0.141	0.126	0.026	0.087	0.113
0.10	LM	PC	0.254	0.288	0.242	0.281	0.256	0.268	0.257	0.256	0.290
0.10	GLS–AR(1)	PC	0.254	0.288	0.242	0.215	0.256	0.268	0.061	0.179	0.259
0.10	iGLS–AR(1)	PC	0.254	0.288	0.242	0.215	0.256	0.268	0.061	0.179	0.259
0.10	iGLS–AR(p)	PC	0.255	0.288	0.242	0.217	0.257	0.268	0.062	0.180	0.260
0.10	LM	BSP	0.254	0.285	0.239	0.280	0.253	0.263	0.258	0.254	0.290
0.10	GLS–AR(1)	BSP	0.255	0.285	0.239	0.210	0.253	0.258	0.053	0.174	0.252
0.10	iGLS–AR(1)	BSP	0.255	0.285	0.239	0.210	0.253	0.258	0.053	0.173	0.251
0.10	iGLS–AR(p)	BSP	0.256	0.285	0.239	0.212	0.254	0.259	0.054	0.174	0.252

Table 4. Mean square prediction errors for different lags $h = 1, 5, 10$. Model (b), $n_B = 1000$
